# Altered miRNA expression patterns in *Tff2* knock-out mice correlate with cellular pathways of neoplastic development and caloric metabolism

**DOI:** 10.3892/ijmm.2012.881

**Published:** 2012-01-10

**Authors:** AFTAB ALI SHAH, PETRA LEIDINGER, ANDREAS KELLER, ANKE WENDSCHLAG, ECKART MEESE, NIKOLAUS BLIN

**Affiliations:** 1 Division of Molecular Genetics, Institute of Human Genetics, University of Tübingen, Tübingen, Germany; 2 Department of Biotechnology, University of Malakand, Chakdara, Pakistan; 3 Institute of Human Genetics, Saarland University, Homburg; 4 Febit Biomed GmbH, Heidelberg; 5 Biomarker Discovery Center, Heidelberg, Germany; 6 Department of Genetics, Medical University, Wroclaw, Poland

**Keywords:** caloric maintenance, gastrointestinal tract, knock-out mouse, microRNA, transcriptional profiling, trefoil peptides

## Abstract

The trefoil peptide family, consisting in mammals of three members namely TFF1, 2 and 3, plays a cytoprotective role in epithelial cells of various tissues, mainly in the digestive tract. *Tff1*, *Tff2* or *Tff3* knock-out mouse models developed various kinds of gastrointestinal impairment. microRNAs are known to be novel gene regulators. We aimed to investigate the physiological role of such miRNAs in *Tff2* knock-out mice. Whole miRNome profiling and *in silico* analysis were performed for *Tff2*-KO and WT mice. Our latest data explored the role of miRNAs in the regulatory cascades and molecular processes of *Tff2*^−/−^ mice. As much as 6% of the *Tff2*-KO mice miRNome was significantly dys-regulated. Further *in silico* analysis suggests that the respective dys-regulated part of the miRNome is involved in human pathological processes, including pancreatic, colorectal and basal cell cancer. Additionally, the dys-regulated miRNome targets pathways involved in carbohydrate metabolism and adipocytokine signaling. The latter links deficient caloric maintenance in *Tff2* and previous observation in *Tff3*-KO mice with miRNAs. In summary, our proof-of-concept study indicates that miRNAs may play an important role in the regulatory processes of the trefoil peptide family, especially in the regulation of cancer-related cascades.

## Introduction

The trefoil (TFF) factor family comprises three members, i.e., TFF1, TFF2 and TFF3. They are synthesized and secreted by epithelial mucosa (1–3). TFFs are involved in mechanisms of defense and repair by interacting with mucins, cytoprotection, and anti-inflammatory effect in the gastrointestinal tract by stimulating cell migration and inhibiting apoptosis (4–11). Their expression is rapidly and coordinately up-regulated in a wide variety of mucosal injury (5) and ulcerative conditions of the gastrointestinal tract including Barrett's esophagus (12), gastric and duodenal ulcers (13,14), pancreatic cancer (15,16) as well as in the small and large intestine in Crohn's disease (17). This overexpression of TFFs emphasizes that they are important peptides involved in the maintenance of the gastrointestinal mucosa. TFF2 is found more abundantly during repair in areas of proliferation, while *Tff2*-deficient mice exhibit immune deficiency (18), increased acid secretion and increased susceptibility to NSAID injury (5).

microRNAs are a family of small (17–24 nucleotides) noncoding RNAs that are involved in post-transcriptional gene regulation through binding to the 3′-untranslated region of their target mRNAs (19). An important feature of miRNA is to regulate multiple targets simultaneously making miRNA a crucial regulator in many physiological conditions. We recently showed that a group of deregulated miRNAs in *Tff3* knock-out mouse model might be involved in regulation of an interesting metabolic pathway (20). To further investigate the role of altered miRNA signature in the *Tff2* knock-out (KO) mouse model and the systemic effect of *Tff2* deregulation, we analyzed the expression of known mouse miRNAs (miRBase Version 14, URL: http://www.mirbase.org/) using a whole miRNome microarray analysis. We used blood cells as a starting material because blood-derived miRNA profiling is a well established system in human (21,22) as well as in mouse models due to remarkable stability of these short nucleotides (20). We hypothesized that deregulated miRNAs in *Tff2*-KO mice might be involved in important biological pathways and since the epithelia of the digestive tract also contribute to immunoresponse blood cells (among them T- and B-cells) (23) will likely carry such important molecular information. Despite latest progress in whole miRNome microarray analysis in various systems, no previous study related to the differential expression of miRNAs in *Tff2*-KO mouse model, has been reported so far.

## Materials and methods

### Animals

*Tff2* deficient mice (129/SV) were generated previously (18) and as a control wild-type (WT) (129/SV) mice were bought from Charles River. All animals (n=6 for each genotype) were kept in a specific pathogen-free facility of the University Clinic of Tuebingen in 12 h dark/light cycles and 22˚C. Food and water were accessible *ad libitum*. All efforts were made to minimize the number of animals used to avoid unnecessary suffering. Care and use of the animals and the experimental protocol were reviewed and approved by the regional board for scientific animal experiments in Tuebingen.

### miRNA extraction

Blood from *Tff2*-KO as well as WT mice was collected in RNAprotect^®^ Animal Blood Tubes (Qiagen GmbH, Hilden). About 400–500 μl of peripheral blood were collected from each animal. After centrifugation of the blood samples at 5,000 × g for 10 min at room temperature (RT), the supernatant was discarded while the pellet was resuspended in 5 ml RNasefree water. Following second centrifugation step at 5,000 × g for 10 min and RT, isolation of total-RNA including miRNA was performed using the miRNeasy kit (Qiagen GmbH). Therefore the blood cell pellet was resuspended in 700 μl QIAzol lysis reagent and incubated for 5 min at RT. A total of 140 μl chloroform was added, vortexed for 15 sec, and incubated for 2–3 min at RT. All samples were centrifuged at 14,000 rpm and 4˚C for 15 min. The RNA in the upper, watery phase was precipitated with 1.5 volume of 100% ethanol. Aliquots of 700 μl of this mixture were placed on a column and centrifuged at 13,000 rpm at RT for 15 sec. After the mixture had completely passed the column, 700 μl of buffer RWT was added to each column, and again centrifuged at 13,000 rpm at RT for 15 sec. Buffer RPE of 500 μl was added to the column and centrifuged at 13,000 rpm at RT for 15 sec. After this step, further 500 μl of buffer RPE was added to the column and centrifuged at 13,000 rpm at RT or 2 min. To dry the column it was centrifuged at 13,000 rpm and RT for 1 min. The RNA was eluted twice with 20 μl RNasefree water by centrifuging at 13,000 rpm at RT for 1 min. The eluted RNA was stored at −70˚C.

### miRNA microarray screening

We analyzed all RNA samples using the Geniom Realtime Analyzer (GRTA, Febit Biomed GmbH, Heidelberg, Germany) and the Geniom Biochip miRNA *Mus musculus*. Each array contains 7 replicates of 710 miRNAs and miRNA star sequences as annotated in the Sanger miRBase version 14.0 (http://microrna.sanger.ac.uk/sequences/) (24). After microarray hybridization for 16 h at 42˚C sample labeling was carried out with biotin using micro-fluidic-based enzymatic on-chip labeling of miRNAs (MPEA) (25). Washing and signal enhancement was processed automatically in the GRTA.

### Expression data and bioinformatics analyses

Geniom Wizard Software was used for microarray evaluation. After that the median signal intensity was extracted for each miRNA and each array from the raw data file such that for each miRNA, seven intensity values have been calculated corresponding to each replicate copy on the array. Next to the background correction, the seven replicate intensity values of each miRNA were summarized by their median value. Quantile normalization was applied to normalize the data across different arrays (26), and all further analyses were carried out using the normalized and background subtracted intensity values. The microarray data were deposited in the publically available database Gene Expression Omnibus (GSE25815).

GeneTrail is a web-based application used for statistical evaluation of high-throughput genomic or proteomic data sets with respect to a reference set. GeneTrail's statistics module includes a novel dynamic-programming algorithm that improves the P-value computation of GSEA (Gene Set Enrichment Analysis) methods considerably. GeneTrail supports many biological categories (KEGG, TRANSPATH, TRANSFAC and GO) (27–29).

Cytoscape program (http://cytoscapeweb.cytoscape.org) was used to visualize the correlations of graphically depicting the regulation of the mRNA targets of the most interesting up-regulated mmu-miR-669c, mmu-miR-329, and down-regulated mmu-miR-688, mmu-miR-30c-1^*^, mmu-miR-201, mmu-miR-761, mmu-miR-715 microRNAs in *Tff2*-KO mice in a convenient way. Nodes represent the pathways, genes and miRNAs while edges show the respective connections (Fig. 3).

### Quantitative real-time PCR

In order to validate microarray results of deregulated miRNAs, we analyzed by qRT-PCR, the expression of some mature miRNAs in total-RNA extracted from all *Tff2*-KO and WT mice. RNA of 10 ng was converted into cDNA using miRNA RT specific primers and TaqMan^®^ microRNA Reverse Transcription kit (Applied Biosystems). The qRT-PCR reactions were performed on Applied Biosystems 7300 Real-Time PCR system using miRNA-specific TaqMan^®^ microRNA Assays (Applied Biosystems). The master mix TaqMan^®^ Universal PCR Master Mix, No AmpErase^®^ UNG (Applied Biosystems) was used for all qRT-PCR reactions according to manufacturer's instructions. A cDNA pool of 6 WT mice was used as a calibrator in the present study. As an endogenous control RNA we used snoRNA202 (small nucleolar RNA, Applied Biosystems), which is commonly used as a control RNA for miRNA studies. miRNA fold changes between the groups were calculated by the delta Ct method.

## Results

### Altered miRNA expression in Tff2 knock-out mice

Among the three mammalian *Tffs*, *Tff2* deserves special attention because of its multiple roles in crucial physiological processes. We aimed at determining whether transcriptional profiles of miRNA are involved in regulating *Tff2* gene activity. Thus, genetically impaired *Tff2* mice were compared with their WT counterparts by microarray screening of miRNAs. In our *Tff2*-KO mouse model a total of 48 miRNAs were detected as differentially expressed. Among those 26 (54%) were down-regulated while 22 (46%) were up-regulated (Table I).

To confirm that the expression of deregulated miRNAs occurred uniformly in all studied animal samples we additionally computed the receiver-operator characteristics curves (ROC) for each of the miRNAs together with the area under the receiver-operator characteristics curve (AUC). ROC shows the sensitivity as function of one minus the specificity. AUC values can range from 0 to 1. An AUC of 0.5 for a miRNA means that the distribution of intensity values generated by RNA from blood of *Tff2*-KO and WT mice cannot be distinguished. The more the AUC differs from 0.5 approaching the values of 0 or 1 the better the miRNA is suited to differentiate between KO and WT. The most extreme values of the AUC are 0 and 1 and correspond to a perfect separation. Out of the 48 significantly deregulated miRNAs, 26 miRNAs had an AUC value above 0.5 (higher median expression in WT than in KO mice) and 22 miRNAs had an AUC value <0.5 (lower median expression in WT than in KO mice).

**Figure 1:**
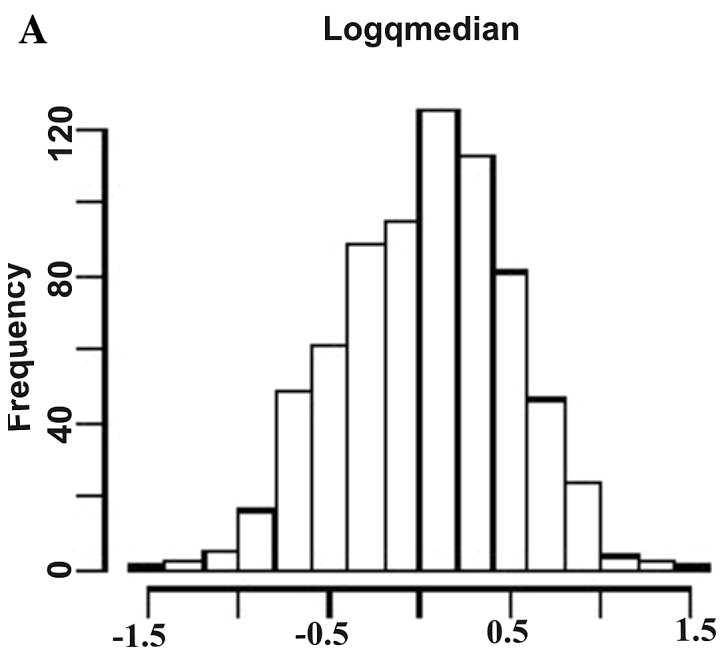

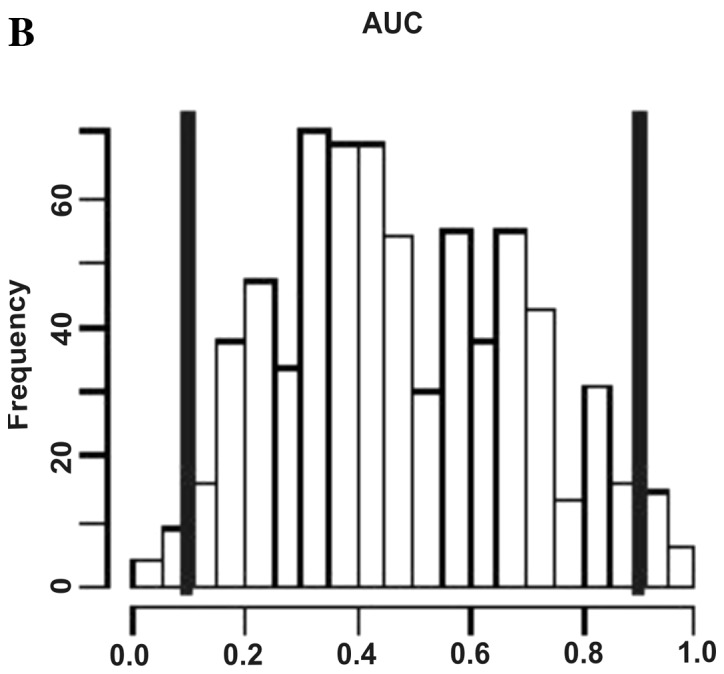

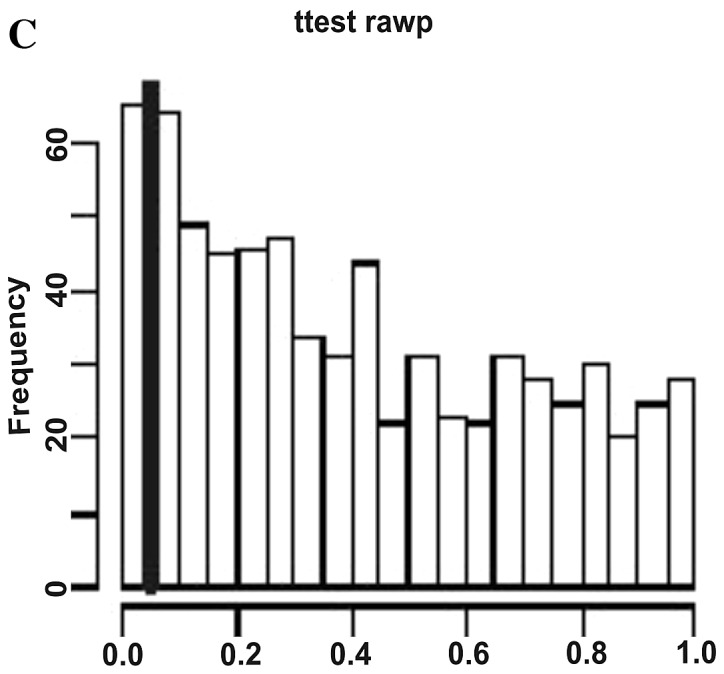
Histogram plots for all screened miRNAs (microarray containing 710 miRNAs and miRNA star sequences in 7 replicates). (A) Shows bars representing miRNA groups arranged from −1.5 to +1.5 (logarithmic scale with 0 indicating the least and ±1.5 the highest values). (B) Histogram plot of the area under the receiver operator characteristics curve (AUC) values shows that only a few miRNAs have AUC values near 1 or 0 indicated by the black bar, while most of the miRNAs have AUC values around 0.5. (C) Histogram plot of the P-values for t-test shows that only few miRNAs have P<0.05 (black bar).

The histogram plots in Fig. 1 show the distribution of logarithm of fold changes (Fig. 1A), AUC values (Fig. 1B), and raw t-test P-values (Fig. 1C) demonstrating a significant differential expression of the deregulated miRNAs.

### Validation of miRNA expression profile by quantitative PCR

**Figure 2:**
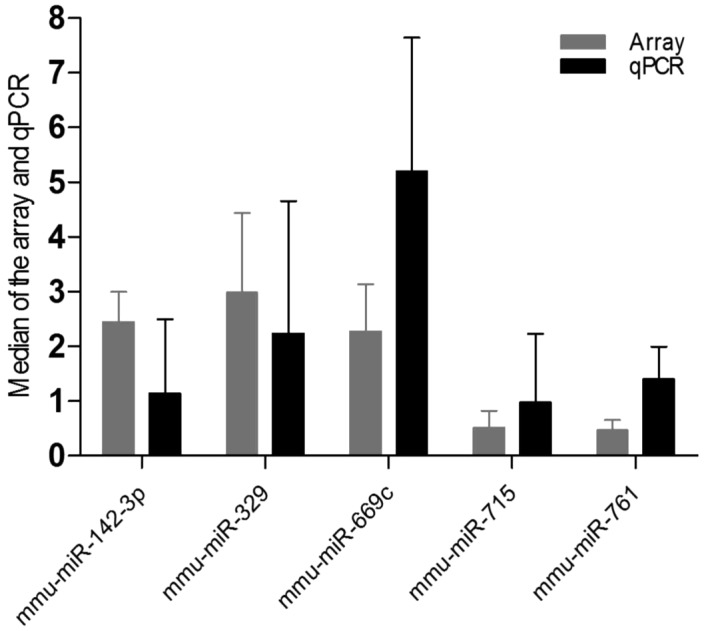
Comparison of the expression levels of 5 deregulated miRNAs in *Tff2*-KO mice as measured by microarray and qPCR. The median of the values (6 mice each replicates 3 times) are shown with standard deviation.

Our microarray screen identified 48 differentially expressed miRNAs in *Tff2*-KO vs. WT mice. To validate these data we analyzed the expression of two down- and three up-regulated miRNAs as a model representation of the whole set (Table I) by qRT-PCR in all *Tff2*-KO and WT samples (Fig. 2). The qRT-PCR results and the array data displayed comparable values thus supporting the original observation.

### In silico analysis of miRNA and their putative target pathways

The above mentioned results prompted us to test whether the collection of deregulated miRNAs is connected to any pathological conditions. We applied a bioinformatic resource for miRNAs target genes to identify possible mRNA interaction networks that are responsible for various cellular processes. This approach using GeneTrail (see Materials and methods for details) provides useful information on the function of microRNA in physiological and pathological conditions.

**Figure 3:**
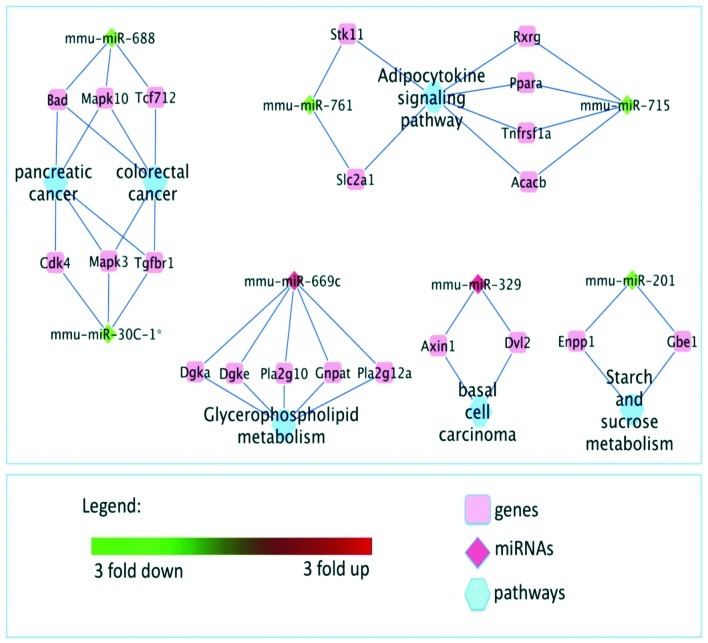
Selected deregulated miRNAs their target genes and pathways involved as indicated by GeneTrail prediction.

We focused our analysis on KEGG (Kyoto Encyclopedia of Genes and Genomes) pathways. We compared the set of the noted up- and down-regulated miRNAs between *Tff2*-KO compared to WT mice to the set of all mouse genes using GeneTrail's standard parameters for the prediction of signaling pathways possibly regulated by these miRNAs. We identified interesting statistically significant signaling pathways (Table II) regulated by selected deregulated miRNAs (Fig. 3). Briefly, we found that mmu-miR-688 and mmu-miR-30c-1^*^ targeting *Tcf712* and *Cdk4* are involved in colorectal and pancreatic cancer, respectively, while the same miRNAs targeting *Bad, Mapk10, Mapk3* and *Tgfbr1* are involved both in pancreatic as well as colorectal cancer. Similarly, mmu-miR-329 (targeting *Axin1* and *Dvl2*) is participating in basal cell carcinoma. Further miRNAs with differential expression pattern are connected with energy metabolism. Here, mmu-miR-669c, targeting *Dgka Dgke Pla2g10, Gnpat* and *pla2g12a*, is involved in glycerophospholid and mmu-miR-201 (targeting *Enpp1* and *Gbe1*) is involved in starch and sucrose metabolism. Additionally, both mmu-miR-761 and mmu-miR-715 (targeting *Stk11*, *Slc2a1* and *Rxrg, Ppara, Tnfrsf1a* and *Acacb*) are involved in adipocytokine signaling pathway.

## Discussion

The three trefoil factor peptides (TFF1-3) are involved in maintenance of epithelial function, thus not surprisingly, in mouse models carrying genetic deletions for *Tff1*, *Tff2* or *Tff3* the animals developed various kinds of gastrointestinal impairment (5,30,31). Previously, tumor specific expression patterns of all trefoil peptides were observed in human patients and the TFFs were noted to be over-expressed in inflammatory and ulcerative lesions (3). By *in situ* hybridization Tff transcription was demonstrated in damaged areas of the digestive tract in rodents. Studies of experimental ulcers in rat stomach (32) disclosed that rat *Tff2* is expressed immediately after injury (0.5–2 h), *Tff3* after 48 h and the growth factors EGF and TGF-α even later, stressing the association of TFFs with the start of the restitution and repair processes. This observation also implies a set program of differential Tff gene activation. While all Tff genes are localized tightly to each other, all three display individual promoters with specific transcriptional signals allowing such differential regulation (33,34). Moreover, search for quantitative trait loci in mouse models indicated a trefoil peptide contribution to diabesity (35) or to macronutrient (carbohydrate/fat) intake (36). The latter study demonstrated a 10-fold up-regulation of *Tff3* in congenic B6.CAST17 mice independent of high-fat vs. high carbohydrate diet. Our recent study demonstrated that genetic impairment of *Tff3* has an influence on the expression pattern of regulatory miRNAs, several of them targeting genes in caloric metabolism. Again, not surprisingly, *Tff3*^−/−^ mice show slower build-up of body mass than their WT counterparts (20). Our preliminary data also connect genetic *Tff2* inactivation with impaired fat metabolism. Moreover, a link of Tff's to the immune system through nutritional pathways and enteric microflora was published (37) and in weaning piglets a probiotic trial indicated an increased *Tff2* and *Tff3* expression (38). In the porcine digestive tract, various segments (from duodenum to distal colon) were reported to express varying patterns of miRNAs pointing to the regulatory impact of these small nucleic acids on specific cellular signaling pathways (39).

These data prompted us to search modified patterns of miRNA expression levels and their target genes in *Tff2*^−/−^ mice. Using miRNA microarrays and cellular fractions from whole blood (20) 22 of miRNAs were found to be up-regulated and 26 to be down-regulated thus exceeding the number of 21 deregulated miRNAs in the *Tff3*^−/−^ model. A screen using a bioinformatics tool (GeneTrail) to link *Tff2*^−/−^ specific miRNAs with their target genes disclosed 7 highly significant regulatory miRNAs (P<0.047) connected with either neoplastic development or carbohydrate metabolism. In the former, colorectal, pancreatic and basal cell cancer are prominent, the latter is represented by sugar and starch metabolism and an adipocytokine pathway. It has been demonstrated in completely independent experiments that mmu-miR-715 as well as mmu-miR30C-1^*^ are involved in specific cellular pathways essentially confirming our observation (40,41). These important regulators display the effect of multiplicity, i.e., one miRNA molecule is targeting different genes that can be functional in one common pathway or even involved in different functional networks. Out of those 7 significant miRNAs only miR-715 has its coding sequence localized in the vicinity of the *Tff* gene cluster (mouse chromosome 17). It is linked to the adipocytokine pathway by targeting one TNF family member. In 2010, Panguluri *et al* (40) demonstrated TNF-like weak inducer of apoptosis (TWEAK) to be a member of the TNF superfamily and by *in vitro*, *in vivo* and *in silico* experiments TWEAK to up-regulate miR-715 about 20-fold which in turn is involved in regulating distinct cellular responses. Since TNFα has been implicated as a link between obesity and insulin resistance this circular loop [(TWEAK)-(miR-715)-(TNFrsf1a)] provides some experimental evidence for this particular miRNA's connection with caloric pathway. Even here the position does not constitute a close neighborhood: the distance is about 8.5 Mb. However, no present model requires genetic vicinity for functional coordination and in the *Tff3*^−/−^ situation the coding sequences of regulatory miRNAs also show no particularly close special linkage.

To substantiate whether the selected miRNAs of our model are in fact deregulated by *Tff2* impairment and share these common target genes for the neoplastic and dietary pathways noted in our study, additional experiments are planned. At first, specific miRNA action in cellular models will be monitored and expression pattern of the target genes will be analyzed by qRT-PCR. Finally, adequate transgenic mice will be put to use. These future experiments should further contribute to our understanding of the variable functional aspects of the trefoil peptide family.

Our proof-of-concept study shows that small non-coding RNA molecules (miRNAs) may play an important role in the regulatory processes of the trefoil peptide family. Despite recent progress in miRNome microarray profiling, no previous study has been conducted so far related to the differential expression of miRNAs in the *Tff2*-KO mouse model.

## Figures and Tables

**Table I:** Logarithm of fold quotients, raw t-test P-values and AUC value of each of all 48 significantly deregulated miRNA tested in (n=6) animals of WT and KO group (7 replicates of each miRNA).

miRNA	Median *Tff2*^−/−^	Median *Tff2*^+/+^	q-median	Log q-median	t-test rawp	AUC
mmu-miR-688	5.62	7.03	0.24	−1.40	0.07	0.83
mmu-miR-1895	10.80	12.12	0.26	−1.32	0.02	0.88
mmu-miR-590-5p	5.78	6.93	0.31	−1.15	0.01	0.91
mmu-miR-488^*^	4.78	5.87	0.33	−1.09	0.01	0.94
mmu-miR-883a-5p	6.04	7.06	0.35	−1.02	0.00	0.91
mmu-miR-712^*^	7.53	8.53	0.36	−1.00	0.00	0.97
mmu-miR-715	8.47	9.47	0.36	−0.99	0.00	0.91
mmu-miR-1954	6.96	7.93	0.37	−0.97	0.00	1
mmu-miR-1907	7.05	8.01	0.38	−0.96	0.02	0.94
mmu-miR-490	5.403	6.34	0.39	−0.93	0.03	0.91
mmu-miR-1946b	7.45	8.38	0.39	−0.92	0.00	0.91
mmu-miR-1899	5.69	6.61	0.39	−0.92	0.00	1
mmu-miR-337-5p	5.95	6.87	0.39	−0.92	0.02	0.86
mmu-miR-761	7.45	8.36	0.40	−0.90	0.00	0.94
mmu-miR-201	6.00	6.84	0.43	−0.84	0.01	1
mmu-miR-669j	7.55	8.36	0.44	−0.80	0.02	0.94
mmu-miR-30c-1^*^	5.16	5.96	0.44	−0.80	0.01	0.94
mmu-miR-1906	8.19	8.99	0.44	−0.79	0.03	0.86
mmu-miR-33	5.45	6.21	0.46	−0.76	0.04	0.86
mmu-miR-297b-3p	5.93	6.67	0.47	−0.74	0.03	0.91
mmu-miR-689	7.07	7.82	0.47	−0.47	0.06	0.83
mmu-miR-719	7.05	7.78	0.48	−0.73	0.02	0.97
mmu-miR-200a	6.07	6.79	0.48	−0.72	0.00	0.94
mmu-miR-298	6.13	6.85	0.48	−0.71	0.11	0.86
mmu-miR-879	6.00	6.71	0.49	−0.70	0.10	0.83
mmu-miR-1928	5.95	6.65	0.49	−0.69	0.07	0.83
mmu-miR-207	5.54	4.84	2.01	0.70	0.24	0.22
mmu-miR-1-2-as	4.56	3.84	2.04	0.71	0.08	0.19
mmu-miR-1982^*^	6.15	5.42	2.07	0.72	0.03	0.11
mmu-miR-744	11.58	10.84	2.08	0.73	0.01	0.05
mmu-miR-1839-5p	7.21	6.45	2.14	0.76	0.03	0.13
mmu-miR-194	11.81	11.01	2.23	0.80	0.15	0.16
mmu-miR-20b	10.20	9.39	2.24	0.80	0.08	0.19
mmu-miR-465b-5p	5.69	4.86	2.28	0.82	0.12	0.16
mmu-miR-151-5p	10.69	9.84	2.34	0.85	0.02	0.11
mmu-miR-1892	7.93	7.07	2.36	0.86	0.03	0.13
mmu-miR-185	11.68	10.81	2.38	0.86	0.06	0.13
mmu-miR-674^*^	8.85	7.97	2.43	0.88	0.00	0.02
mmu-miR-142-3p	5.17	4.25	2.50	0.91	0.00	0.02
mmu-miR-1894-3p	8.31	7.39	2.52	0.92	0.04	0.19
mmu-miR-669c	9.22	8.29	2.53	0.92	0.02	0.05
mmu-miR-99b	8.56	7.63	2.53	0.93	0.02	0.08
mmu-miR-7a^*^	9.01	8.07	2.57	0.94	0.03	0.13
mmu-let-7g	9.40	8.41	2.69	0.99	0.15	0.22
mmu-miR-329	6.58	5.48	3.00	1.09	0.09	0.16
mmu-mmu-let-7e	7.97	6.82	3.16	1.15	0.12	0.19
mmu-miR-195	11.55	10.39	3.21	1.16	0.00	0.05
mmu-miR-125a-5p	8.97	7.72	3.50	1.25	0.00	0.08

**Table II:** The 7 deregulated miRNAs with statistically significant (P<0.05) target genes and their pathways. The listed miRNAs target genes of a particular pathway (multiplicity), the gene names are shown on the right.

miRNAs	Subcategory name	P-value	Observed number of genes	GeneIDs of test set in subcategory
mmu-miR-688	Colorectal cancer	0.016	3	Bad, Mapk10, Tcf712
	Pancreatic cancer	0.047	2	Bad, Mapk10
mmu-miR-30c-1^*^	Colorectal cancer	0.042	2	Tgfbr1, Mapk3
	Pancreatic cancer	0.012	3	Tgfbr1, Mapk3, Cdk4
mmu-miR-329	Basal cell carcinoma	0.024	2	Axin1, Dvl2
mmu-miR-669c	Glycerophospholipid metabolism	0.016	5	Dgka, Dgke, Pla2g10, Gnpat, Pla2g12a
mmu-miR-201	Starch and sucrose metabolism	0.020	2	Enpp1, Gbe1
mmu-miR-715	Adipocytokine signaling pathway	0.011	4	Acacb, Ppara, Rxrg, Tnfrsf1a
mmu-miR-761	Adipocytokine signaling pathway	0.028	2	Slc2a1, Stk11
